# Importance of Early Treatment for the Insane

**Published:** 1885-10

**Authors:** 


### IMPORTANCE OF EARLY TREATMENT FOR THE INSANE.

The existence of mental derangement among people of all ranks and conditions in life calls for treatment in like manner as physical disease. According to the origin of the disorder, measures for its correction may be addressed more especially to the mind or the body; and, indeed, there is such a reciprocal action and reaction between the spiritual and corporeal elements, which go to make up that compound being known as man, that one part can scarcely be disturbed without, to some extent, affecting the other. It is therefore important, in providing for the relief of mental troubles, that all the hygienic precautions looking to physical health shall receive due consideration. The great advances made in these latter years in the plans of treating the insane are to be reckoned among the indications of progress in science, based upon a close observation of cause and effect, so that the remnant of right reason may be turned to account in restoring the harmonious action of the intellectual faculties. It was not formerly understood that mental derangement consisted in many cases, not so much of perversion of the entire intellect, as in a want of equilibrium between the various mental faculties. Certain attributes of the mind may be disordered, while others continue in their normal state, and may be made to some extent subservient to the correction of the deranged functions. It is therefore of much importance for the proper management of the disease in its incipiency, that the true nature of the mental disorder should be appreciated; and as only those who have given special attention to the study of insanity can be thoroughly fitted for understanding the phases of lunacy at the outset, those cases which present indications of a derangement of the intellectual faculties, independent of the effects of acute physical diseases, should be submitted at once to their treatment. We know how reluctant families are to have one of their number consigned to a lunatic asylum, under the erroneous impression of its operating as a stigma upon all who are related to the individual by the ties of consanguinity. But if they will recall the history of those who have been restored by hospital care in the early stage of mental derangement, as compared with the few who are cured after the lapse of many months without such care, it should reconcile all concerned to the prompt adoption of this measure. The fact of being insane is a great misfortune to the individual, and a calamity to those associated with the subject of such an affliction; yet it brings no sort of disgrace or dishonor with it, and is not in any way aggravated in its prejudicial influence by a proceeding which relieves the family and friends of the responsibility of treatment. It must be patent to those who have been associated with persons of unsound mind, that the relations of private life with them are hazardous in the extreme, as there may be developed sudden impulses leading to the injury of others or to self-destruction. A public institution for their protection and supervision affords the best security against violence on the part of insane persons, and at the same time presents a better prospect of the success of curative measures. The evils attending the mismanagement of some establishments for mental alienation should not deter people from seeking a hospital conducted upon proper principles for the treatment of mental diseases; and the care of a medical adviser and of nurses for the patient should be viewed as in other hospitals.

No language of condemnation is too strong for any individual or family that should, for a selfish purpose, consign any person to an insane asylum who is in the enjoyment of right reason. A terrible disclosure has just come to the public, in which a young lady, in the possession of all her mental faculties, had been committed many years ago to the guardianship of an institution for lunatics at the North, and had spent the prime of her life in contact with those suffering from mental alienation. It is only to be regretted that the inhuman father, who from politic and interested motives victimized his daughter, did not survive to receive the righteous indignation of every right-minded man and woman throughout the land.

But with such provisions against impositions as exist in most of the hospitals for those of unsound mind throughout the United States, and the honest administration of competent trustees, there is little to fear from this baseness of human depravity. We may rest assured that none will be confined without just and sufficient cause at the present day, and the object of these remarks is to impress upon medical men, and the community generally, the urgent need of proper attention and expert treatment in the developing stage of mental alienation.

